# Supplementary material for: Exploring barriers and facilitators to women’s intention and behavior to seek treatment for distressing sexual problems
Source: PLoS One. 2023 Jul 18;18(7):e0288205. doi: 10.1371/journal.pone.0288205 (PMC10353825; doi:10.1371/journal.pone.0288205)
Supplement: S1 Table — (DOC) [file pone.0288205.s001.doc]

| **S1 Table. Sociodemographic and socioeconomic variables for the main income earner (*N* = 800)** | | | |
| --- | --- | --- | --- |
|  | |  | *n* (%) |
| Participant is the main income earner | | |  |
|  | Yes, participant earns 50% or more of household income | | 287 (35.9) |
|  | Yes, participant is living in a shared flat | | 207 (25.9) |
|  | No, another person earns more than 50% of household income | | 207 (25.9) |
|  | No, participant still lives with parents | | 130 (16.3) |
| School education of main income earner | | |  |
|  | | No high-school degree | 115 (14.4) |
|  | | High-school degree | 685 (85.6) |
| Occupation of main income earner | | |  |
|  | | Freelancer, civil servant, executive employee | 101 (12.6) |
|  | | Self-employed | 53 (6.6) |
|  | | Intermediate civil servant, farmer, qualified employee with authority to issue directives | 96 (12.0) |
|  | | Skilled worker, qualified employee | 224 (28.0) |
|  | | Lower-level civil servant | 12 (1.5) |
|  | | Employee without authority to issue directives | 57 (7.1) |
|  | | Other (e.g., blue-collar worker, student, apprentice, retiree, on parental or maternity leave) | 257 (32.2) |
