# Supplementary material for: Exploring barriers and facilitators to women’s intention and behavior to seek treatment for distressing sexual problems
Source: PLoS One. 2023 Jul 18;18(7):e0288205. doi: 10.1371/journal.pone.0288205 (PMC10353825; doi:10.1371/journal.pone.0288205)
Supplement: S2 Table — (DOC) [file pone.0288205.s002.doc]

| **S2 Table. Explanation of intention to utilize psychological online interventions for sexual problem.** | | | | | | | | | |
| --- | --- | --- | --- | --- | --- | --- | --- | --- | --- |
|  | |  | |  |  |  | 95.0% CI | | |
|  |  | b | SE | β | T | p | Lower | | Upper |
| Constant | | 3.75 | 0.73 |  | 5.13 | < .001 | 2.31 | 5.18 | |
| Personal characteristics | |  |  |  |  |  |  |  | |
|  | Age | -0.01 | 0.01 | -0.05 | -1.31 | .191 | -0.02 | 0.00 | |
|  | Partnership (yes/no) | -0.05 | 0.14 | -0.01 | -0.35 | .724 | -0.32 | 0.22 | |
|  | Urbanicity (rural/urban) | 0.35 | 0.14 | 0.09 | 2.56 | .011 | 0.08 | 0.61 | |
|  | Sexual orientation (other/excl. heterosexual) | 0.05 | 0.13 | 0.01 | 0.34 | .733 | -0.22 | 0.31 | |
|  | Objective socioeconomic status | -0.14 | 0.11 | -0.05 | -1.23 | .218 | -0.36 | 0.08 | |
|  | Subjective socioeconomic status | 0.00 | 0.08 | 0.00 | 0.00 | .997 | -0.15 | 0.15 | |
|  | Depression and anxiety (PHQ-4) | -0.04 | 0.02 | -0.07 | -1.77 | .078 | -0.09 | 0.00 | |
|  | Any prior help for sexual problems | 0.13 | 0.14 | 0.04 | 0.92 | .358 | -0.15 | 0.41 | |
| Sexual problem symptoms | |  |  |  |  |  |  |  | |
|  | Sexual distress (SDS-SF) | 0.03 | 0.02 | 0.08 | 1.50 | .135 | -0.01 | 0.07 | |
|  | Low desire (SCS-W) | -0.05 | 0.08 | -0.03 | -0.59 | .552 | -0.21 | 0.11 | |
|  | Lack of arousal (SCS-W) | 0.04 | 0.10 | 0.02 | 0.38 | .707 | -0.17 | 0.24 | |
|  | Difficulties reaching Orgasm (SCS-W) | -0.07 | 0.06 | -0.05 | -1.16 | .246 | -0.20 | 0.05 | |
|  | Painful Intercourse (SCS-W) | -0.12 | 0.08 | -0.06 | -1.41 | .158 | -0.28 | 0.05 | |
| Cognitive factors | |  |  |  |  |  |  |  | |
|  | Self-stigma for seeking help (SSOSH-S) | -0.04 | 0.01 | -0.16 | -3.92 | < .001 | -0.05 | -0.02 | |
|  | Sexual self-efficacy (MSSCQ) | 0.09 | 0.13 | 0.04 | 0.68 | .495 | -0.17 | 0.36 | |
|  | Sexual assertiveness (MSSCQ) | 0.05 | 0.10 | 0.03 | 0.46 | .642 | -0.15 | 0.24 | |
|  | Sexual problem management (MSSCQ) | 0.06 | 0.10 | 0.02 | 0.57 | .568 | -0.14 | 0.25 | |
|  | Sexual esteem (MSSCQ) | -0.17 | 0.13 | -0.09 | -1.26 | .206 | -0.43 | 0.09 | |
| MSSCQ = Multidimensional Sexual Self-Concept Questionnaire. PHQ-4 = Patient Health Questionnaire 4. SCS-W = Sexual Complaints Screener for Women. SDS-SF = Sexual Distress Scale Short Form. SSoSH-S = Self-Stigma of Seeking Help Scale – Sexual | | | | | | | | | |
